# First Admission Neutrophil–Lymphocyte Ratio May Indicate Acute Prognosis of Ischemic Stroke

**DOI:** 10.5041/RMMJ.10440

**Published:** 2021-07-20

**Authors:** Murat Alpua, Bahar Say, Ilknur Yardimci, Ufuk Ergün, Ucler Kisa, Ozlem Doğan Ceylan

**Affiliations:** 1 Department of Neurology, Kirikkale University, Faculty of Medicine, Kirikkale, Turkey; 2 Department of Biochemistry, Kirikkale University, Faculty of Medicine, Kirikkale, Turkey; 3 Department of Medical Biochemistry, Ankara University, Faculty of Medicine, Ankara, Turkey

**Keywords:** Ischemic stroke, periostin, prognosis, scale

## Abstract

**Objectives:**

Our study aimed to determine the relationship between serum periostin levels, and the neutrophil–lymphocyte ratio (NLR) with ischemic stroke subtypes, clinical stroke scales, and acute prognosis in patients with acute ischemic stroke.

**Materials and Methods:**

Forty-two ischemic stroke patients and 39 age- and sex-matched healthy volunteers were included in our study. Demographic characteristics including age and gender were recorded. Blood serum periostin and NLR values were evaluated in the first 24 hours after admission. Serum periostin levels were compared with healthy controls of similar age and sex. Lesion localization was determined by cranial CT or diffusion MRI of the patients. Stroke scales were recorded on days 1 and 7 of hospitalization in the study group.

**Results:**

The mean serum periostin levels were higher than in the control group, but no statistically significant difference was found. There was no correlation between serum periostin levels and prognosis of stroke. First admission NLRs were statistically higher than in the control group. The first admission NLRs were positively correlated with the first admission National Institute of Health Stroke Scale score and the day 7 modified Rankin score.

**Conclusion:**

Our study is the first study to evaluate both NLR and serum periostin levels in all types of acute ischemic stroke. While our study did not show that first admission serum periostin levels can be used as a biomarker in ischemic stroke, it did indicate that the first admission NLR can be used for acute prognosis of ischemic stroke.

## INTRODUCTION

Stroke is the third leading cause of death following coronary heart disease and all cancers in both developed countries and worldwide. The average global stroke incidence annually is two new strokes per 1,000 people.^1^ About 80% of all stroke cases are ischemic stroke. The Bamford classification system is an important tool for evaluating ischemic stroke. Based on clinical findings, patients are divided into four subgroups: partial anterior circulation infarcts, total anterior circulation infarcts, lacunar infarcts, and posterior circulation infarction. Several factors impact life prediction after stroke. In ischemic stroke patients, the survival rate during the first 30 days is 85%, whereas in hemorrhagic stroke, this rate ranges from 20% to 52%. Mortality is higher in hemorrhagic stroke patients in the first 30 days, whereas mortality increases in ischemic stroke patients over time. Lesion presence in vital areas, especially the brain stem, supratentorial region, and insula region, is associated with higher mortality. However, there is no definitive information about the prognosis of post-stroke recovery. Therefore, many biomarkers have been researched to predict the prognosis of stroke, although very few biomarkers are currently being used for follow-up of stroke patients in daily clinical practice.

Periostin (molecular weight, 90 kD) is an extracellularly secreted, structural adhesion molecule that is similar to fasciclin I. It was first detected in osteoblast cells but also exists in mesenchymal tissues of other organs.^2^ The use of periostin as a biomarker has been investigated in many different diseases, including for acute vascular diseases of the central nervous system. For example, it has been suggested that increased serum periostin levels can be used as a biomarker for poor prognoses in subarachnoid hemorrhages.^3^ Similarly, it has been suggested that increased periostin levels may correlate with disease severity in stroke cases associated with large-artery atherosclerosis.^4^

The neutrophil–lymphocyte ratio (NLR) has been considered as an easy and practical measure that provides valuable information in the diagnosis and prognosis of various diseases in recent years. Several studies have shown that the NLR can also be used as a prognostic marker in acute stroke.^5^,^6^

In the current study, we aimed to determine the relationship between serum periostin levels and NLR with ischemic stroke subtypes, clinical stroke scales, and acute stage prognosis in patients with acute ischemic stroke. Our study is the first study to evaluate both NLR and serum periostin levels in all types of acute ischemic stroke.

## METHODS

Our study was carried out between December 2018 and February 2019 in Kirikkale University Faculty of Medicine Neurology Clinic. All procedures performed in studies involving human participants were in accordance with the ethical standards of the institutional and/or national research committee and with the 1964 Helsinki declaration and its later amendments or comparable ethical standards. Approval from the institutions’ ethical committee was obtained, and the protocol of the study was explained to the patients and patients’ relatives. After receiving detailed information, each patient or relative signed an informed consent form.

We prospectively evaluated 42 patients who underwent a neurological examination, were diagnosed with ischemic cerebrovascular disease by neuroradiological examination, and were admitted to our hospital for follow-up and treatment. To the extent possible, sex- and age-matched healthy volunteer workers, without a history of any diseases, were taken as controls. Previous similar studies were also taken into consideration.

Patients with the following were excluded from the study: previous cerebrovascular accident, history of head trauma, current uremia; cirrhosis, malignancy, chronic heart disease, chronic lung disease, transient ischemic attack, epidural, subdural hematoma, subarachnoid hemorrhage.

Ischemic stroke patients were analyzed for stroke sub-type based on the Bamford classification system, as described above.

### Stroke Severity Evaluation

An important predictor of stroke survival is lesion severity. Several studies have shown that life expectancy in severe lesions is low.^7^,^8^ Many scales have been developed to describe neurological dysfunction in patients with ischemic stroke. These scales are used to gain insight into patient prognosis, and to aid in the decision-making process regarding optimal therapy and follow-up of patients. The scales most commonly used for this purpose are the National Institute of Health Stroke Scale (NIHSS) and the modified Rankin Scale (mRS).

The NIHSS is the most commonly used scale for determining lesion severity in the early period after the stroke. Patients are evaluated in 11 categories (consciousness, language, dysarthria, eye movements, visual field, neglect, facial paresis, proximal arm and leg force, extremity ataxia, and sensation). The total score is between 0 and 42, with each category scored as 0–2 or 0–4. A total of 0–6 points is considered mild; 7–15, moderate; and 16 or higher, severe; the higher the score, the greater stroke severity. A good prognosis estimation will reduce the incidence of wrong treatment and reduce the cost by optimal use of available facilities.

In terms of patient follow-up, the mRS range is 0–5 points; the higher the score, the greater the disability: patients receiving 1 or 2 points are independent, whereas those with 3 or more points remain with moderate (3 points) to severe (5 points) disability. A score of 6 indicates that the patient is dead.

For our study, both the NIHSS and mRS scales were used to monitor patient prognosis and then compared to their biomarkers. Patient scores were recorded on the first and seventh days after hospital admission.

### Clinical Evaluation and Analyses

Age, sex, NLR, neurological examinations, and radiological imaging (CT and MRI) results of the patients were recorded.

Intravenous blood samples were collected from both patients and controls. Blood samples were taken from patients within the first 24 hours of symptoms onset. Serum was separated from the blood samples by centrifugation at 1000*g* for 20 min and was kept frozen at −80°C until used for analyses to measure periostin levels. Serum periostin levels were measured using the Human Periostin ELISA Kit (Cloud-Clone Corp., Katy, TX, USA) with the enzyme-labeled immunometric assay method (detection range: 0.156–10 ng/mL).

SPSS version 16.0 was used to analyze the results. A *P*-value <0.05 was considered to indicate statistical significance. Categorical variables were expressed as proportions. Continuous variables were presented as mean±SD or medians and ranges. The chi-square test was used to test differences in categorical values and the Student *t* test for continuous variables. Relationships among periostin, NLR with stroke scales, and stroke subtypes were examined using Spearman’s correlation in all subjects.

## RESULTS

**Figure 1:**
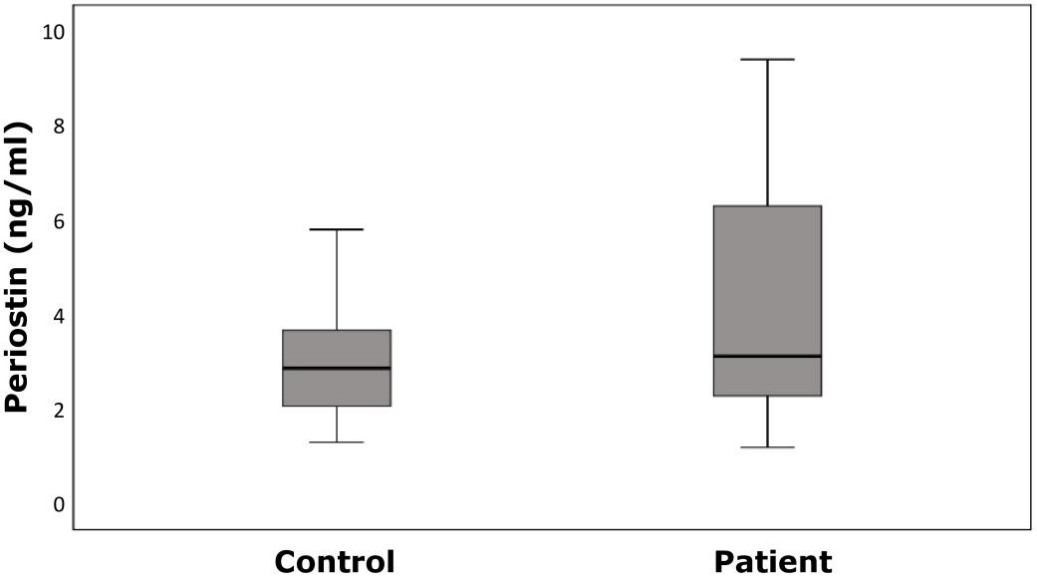
Periostin Levels in the Patient and Control Groups. Data distribution is shown in the box-plot. The lowest point is the minimum of the data set, and the highest point is the maximum of the data set. The box is drawn from *Q_1_* to *Q_3_* with the horizontal line denoting the median.

**Figure 2:**
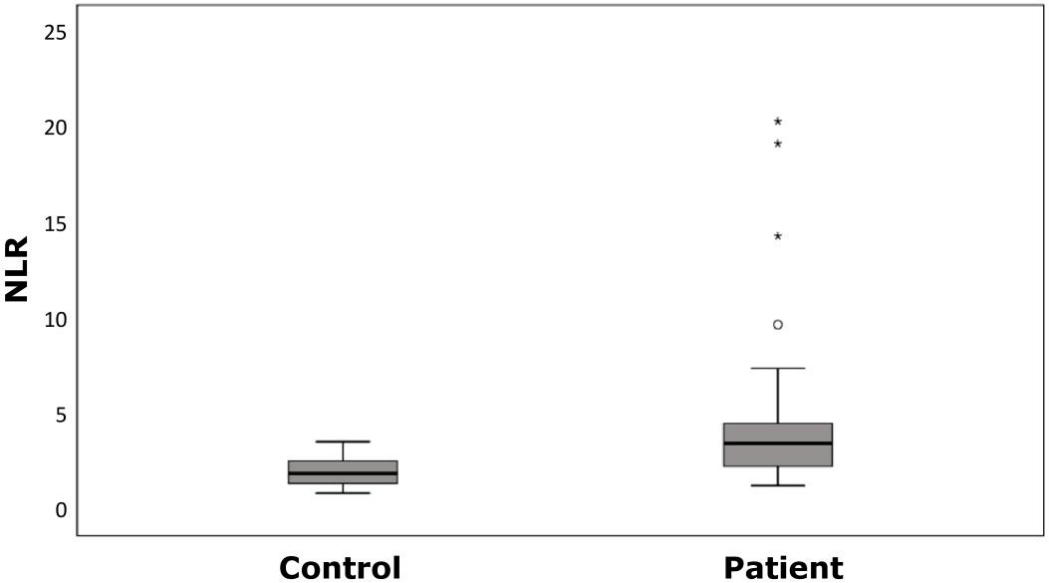
Neutrophil–Lymphocyte Ratio (NLR) Levels in the Patient and Control Groups. Data distribution is shown in the box-plot. The lowest point is the minimum of the data set, and the highest point is the maximum of the data set. The box is drawn from *Q_1_* to *Q_3_* with the horizontal line denoting the median. The circle and asterisk represent the mild and extreme outliers in the data, respectively.

Thirty (71.4%) male and 12 female (28.6%) patients with acute ischemic stroke were included in the study, mean age 64.6±14.6 years. There were no significant differences between the groups in terms of age and gender (Table 1). Serum periostin levels were higher in the patient group compared to the healthy group, but with no statistically significant difference (Figure 1). The NLRs were statistically significantly higher in the patient group compared to the healthy group (Table 1, Figure 2). Concomitant diseases such as hypertension or diabetes mellitus were present in 57.1% of the patients. Total anterior circulation infarcts accounted for only 7.1% of the patients (Table 2). No correlation was found between the accompanying diseases and serum periostin and NLRs (Table 3).

**Table 1:** Features of Patient and Control Group.

Variable	Control (*n*=40)	Patient (*n*=42)	*P*
Mean age, y (SD)	68.4 (8.8)	64.6 (14.6)	0.161*
Female sex, *n* (%)	15 (37.5)	12 (28.6)	0.532†
Median periostin, ng/ml [range]	2.87 [1.3–5.8]	3.12 [1.19–9.4]	0.091‡
Median NLR [range]	1.87 [0.85–3.53]	3.45 [1.25–20.3]	<0.001‡

* Independent samples *t* test.

† Chi-square test.

‡ Mann–Whitney *U* test.

NLR, neutrophil–lymphocyte ratio.

**Table 2:** Patient Group Characteristics.

Characteristics	Value
Comorbidity, *n* (%)	
None	18 (42.9)
HT	9 (21.4)
DM	4 (9.5)
HT+DM	11 (26.2)

Bamford classification, *n* (%)	
TACI	3 (7.1)
PACI	15 (35.7)
POCI	9 (21.4)
LACI	15 (35.7)

Median NIHSS [range]	
At admission	2 [1–20]
At 7th day	1 [0–25]

Median mRS [range]	
At admission	2 [1–5]
At 7th day	1 [0–6]

DM, diabetes mellitus; HT, hypertension; LACI, lacunar infarcts; mRS, modified Rankin Scale; NIHSS, National Institute of Health Stroke Scale; PACI, partial anterior circulation infarcts; POCI, posterior circulation infarction; TACI, total anterior circulation infarcts.

**Table 3:** Possible Associations between Either Periostin or NLR and Stroke Severity in the Patient Group.

Factors	Periostin		NLR	

	Correlation Coefficient	*P*	Correlation Coefficient	*P*
NIHSS (at admission)	0.055	0.729	0.307	0.048

NIHSS (at 7th day)	−0.150	0.342	0.281	0.072

mRS (at admission)	0.090	0.572	0.236	0.132

mRS (at 7th day)	−0.115	0.469	0.315	0.042

**Factors**	**Median Periostin, ng/ml [range]**	***P***	**Median NLR [range]**	***P***

Comorbidity				
None	3.88 [1.56–9.4]	0.731	3.6 [1.65–14.3]	0.448
HT	3.12 [1.19–7.18]		3.41 [2.21–19.13]	
DM	2.94 [1.58–6.26]		2.95 [1.29–4.49]	
HT+DM	2.87 [1.42–7.41]		2.52 [1.25–20.3]	

Bamford classification				
TACI	6.51 [1.97–9.4]	0.514	4.42 [3.46–20.3]	0.500
PACI	3.12 [1.42–7.41]		3.5 [1.25–19.13]	
POCI	2.75 [1.19–5.44]		2.61 [1.65–14.3]	
LACI	3.26 [1.56–9.13]		3.43 [1.29–7.37]	

DM, diabetes mellitus; HT, hypertension; LACI, lacunar infarcts; mRS, modified Rankin Scale; NIHSS, National Institute of Health Stroke Scale; PACI, partial anterior circulation infarcts; POCI, posterior circulation infarction; TACI, total anterior circulation infarcts.

## DISCUSSION

In recent years, there has been a growing interest in the analysis of neurobiological markers due to brain damage in various central nervous system diseases. A variety of biomarkers have been studied in stroke, which is a common disease of the central nervous system. However, there is still no serum biomarker that can be used in clinical practice for stroke diagnosis and follow-up. Periostin is a 90-kDa extracellular matrix protein which belongs to the fasciclin family. The periostin molecule plays an important role in the initiation of inflammation,^9^ and its use as a biomarker, particularly in inflammatory diseases, has been the subject of several studies. Animal models have shown that periostin levels increase after ischemic stroke.^10^,^11^ The biomarker and prognostic role of periostin have also been studied in stroke in humans by He et al., but only for large-artery strokes.^4^ In their study, increased serum periostin levels on day 6 after the stroke were shown to be associated with poor prognosis in patients with major artery stroke. However, our study included all stroke groups, and serum periostin levels were not correlated with acute prognosis. In our study, this situation may be explained by the small number of patients with large lesions (7%) and by the fact that we only considered periostin levels at 24 hours. In addition, and consistent with the literature, our study found that first admission serum periostin levels were not statistically different from the control group. However, another study showed that serum periostin levels were elevated early at 6 hours after the event in intracerebral hemorrhages.^12^ In our study, although there was no statistically significant difference, patient serum periostin levels were found to be higher than the control group at 24 hours, and existing different ischemic stroke subtypes may explain this result in our study.

The NLR is an important and easily applicable biomarker, especially for inflammatory diseases. In recent years, many studies have been conducted on its use as a biomarker in vascular diseases^13^; it has also been suggested that NLR could be used as a prognostic indicator in large-artery strokes.^14^ In our study, which included all stroke groups according to the Bamford classification, the first admission NLRs were significantly higher in patients than controls. This indicates that NLR can be used as a biomarker in all ischemic stroke groups.

Previous studies have shown that high NLR is associated with both stroke severity and a short-term poor prognosis.^15^,^16^ Our study demonstrated a positive correlation between NLR and first admission NIHSS and day 7 mRS, thereby suggesting that NLR can be used for acute prognosis in all ischemic stroke subgroups.

Since concomitant systemic diseases such as diabetes mellitus and hypertension are important for prognosis of stroke patients, the candidate biomarkers should be evaluated in this respect. To date, no studies have been published investigating the relationship between periostin and hypertension, although a recent study has shown that serum periostin levels are low in diabetic patients.^17^ Nevertheless, it has been suggested that higher NLRs may be a predictive biomarker for diabetes mellitus,^18^ and recent studies have shown that the NLR is higher in hypertensive patients than in the normal population.^19^ Our study showed no statistically significant correlation between accompanying diseases and existing biomarkers.

There were some limitations related to our study. First, the study group was not very large. Secondly, we used only the patient serum periostin and NLR values taken at the time of first admission. Thirdly, while there were comorbidities in the patient group, the control group consisted of completely healthy individuals. However, our study showed that comorbidities such as hypertension and diabetes mellitus did not affect periostin and NLR values.

In conclusion, our current study showed that NLR may be a diagnostic and prognostic biomarker in all subtypes of acute ischemic stroke. Larger-scale studies should be performed to evaluate the usefulness of serum periostin levels as a diagnostic biomarker in all subtypes of acute ischemic stroke.

## Abbreviations

NIHSS: National Institute of Health Stroke Scale
NLR: neutrophil–lymphocyte ratio

## References

[1] Alpua M, Bakır F, Oztekin N (2014). The relationship between serum S100B protein levels and lesion type, size and location in patients with acute cerebrovascular stroke [Turkish]. KÜ Tıp Fak Derg.

[2] Parulekar AD, Atik MA, Hanania NA (2014). Periostin, a novel biomarker of TH2-driven asthma. Curr Opin Pulm Med.

[3] Luo W, Wang H, Hu J (2018). Increased concentration of serum periostin is associated with poor outcome of patients with aneurysmal subarachnoid hemorrhage. J Clin Lab Anal.

[4] He X, Bao Y, Shen Y (2018). Longitudinal evaluation of serum periostin levels in patients after large-artery atherosclerotic stroke: a prospective observational study. Sci Rep.

[5] Yu S, Arima H, Bertmar C, Clarke S, Herkes G, Krause M (2018). Neutrophil to lymphocyte ratio and early clinical outcomes in patients with acute ischemic stroke. J Neurol Sci.

[6] Wan J, Wang X, Zhen Y (2020). The predictive role of the neutrophil-lymphocyte ratio in the prognosis of adult patients with stroke. Chin Neurosurg J.

[7] Bjerkreim AT, Khanevski AN, Thomassen L (2019). Five-year readmission and mortality differ by ischemic stroke subtype. J Neurol Sci.

[8] Liu X, Xu G, Wu W, Zhang R, Yin Q, Zhu W (2006). Subtypes and one-year survival of first-ever stroke in Chinese patients: the Nanjing Stroke Registry. Cerebrovasc Dis.

[9] Izuhara K, Nunomura S, Nanri Y (2017). Periostin in inflammation and allergy. Cell Mol Life Sci.

[10] Shimamura M, Taniyama Y, Nakagami H (2014). Long-term expression of periostin during the chronic stage of ischemic stroke in mice. Hypertens Res.

[11] Shimamura M, Taniyama Y, Katsuragi N (2012). Role of central nervous system periostin in cerebral ischemia. Stroke.

[12] W-J Ji, X-M Chou, G-Q Wu (2017). Association between serum periostin concentrations and outcome after acute spontaneous intracerebral hemorrhage. Clin Chim Acta.

[13] Afari ME, Bhat T (2016). Neutrophil to lymphocyte ratio (NLR) and cardiovascular diseases: an update. Expert Rev Cardiovasc Ther.

[14] Goyal N, Tsivgoulis G, Chang JJ (2018). Admission neutrophil-to-lymphocyte ratio as a prognostic biomarker of outcomes in large vessel occlusion strokes. Stroke.

[15] Xue J, Huang W, Chen X (2017). Neutrophil-to-lymphocyte ratio is a prognostic marker in acute ischemic stroke. J Stroke Cerebrovasc Dis.

[16] Zhang J, Ren Q, Song Y (2017). Prognostic role of neutrophil-lymphocyte ratio in patients with acute ischemic stroke. Medicine (Baltimore).

[17] Sharma A, Demissei BG, Tromp J (2017). A network analysis to compare biomarker profiles in patients with and without diabetes mellitus in acute heart failure. Eur J Heart Fail.

[18] Yilmaz H, Ucan B, Sayki M (2014). Usefulness of the neutrophil-to-lymphocyte ratioto prediction of type 2 diabetes mellitus in morbid obesity. Diabetes Metab Syndr.

[19] Wang H, Hu Y, Geng Y (2017). The relationship between neutrophil to lymphocyte ratio and artery stiffness in subtypes of hypertension. J Clin Hypertens (Greenwich).

